# Design and Implementation of a Smart Home System Using Multisensor Data Fusion Technology

**DOI:** 10.3390/s17071631

**Published:** 2017-07-15

**Authors:** Yu-Liang Hsu, Po-Huan Chou, Hsing-Cheng Chang, Shyan-Lung Lin, Shih-Chin Yang, Heng-Yi Su, Chih-Chien Chang, Yuan-Sheng Cheng, Yu-Chen Kuo

**Affiliations:** 1 Department of Automatic Control Engineering, Feng Chia University (FCU), No. 100, Wenhwa Road, Seatwen, Taichung 40724, Taiwan; hcchang@fcu.edu.tw (H.-C.C.); sllin@fcu.edu.tw (S.-L.L.); d0243331@fcu.edu.tw (C.-C.C.); d0243225@fcu.edu.tw (Y.-S.C.); m0429322@fcu.edu.tw (Y.-C.K.); 2 Department of Mechanical and Mechatronics Systems Research Labs., Industrial Technology Research Institute (ITRI), 195, Sec. 4, Chung Hsing Rd., Chutung, Hsinchu 31040, Taiwan; phchou@itri.org.tw; 3 Department of Mechanical Engineering, National Taiwan University (NTU), No. 1, Sec. 4, Roosevelt Road, Taipei 10617, Taiwan; scy99@ntu.edu.tw; 4 Department of Electrical Engineering, Feng Chia University (FCU), No. 100, Wenhwa Road, Seatwen, Taichung 40724, Taiwan; hengyisu@fcu.edu.tw

**Keywords:** wearable intelligent technology, artificial intelligence, sensing data fusion, gesture recognition, indoor positioning, smart energy management, home safety, smart home automation

## Abstract

This paper aims to develop a multisensor data fusion technology-based smart home system by integrating wearable intelligent technology, artificial intelligence, and sensor fusion technology. We have developed the following three systems to create an intelligent smart home environment: (1) a wearable motion sensing device to be placed on residents’ wrists and its corresponding 3D gesture recognition algorithm to implement a convenient automated household appliance control system; (2) a wearable motion sensing device mounted on a resident’s feet and its indoor positioning algorithm to realize an effective indoor pedestrian navigation system for smart energy management; (3) a multisensor circuit module and an intelligent fire detection and alarm algorithm to realize a home safety and fire detection system. In addition, an intelligent monitoring interface is developed to provide in real-time information about the smart home system, such as environmental temperatures, CO concentrations, communicative environmental alarms, household appliance status, human motion signals, and the results of gesture recognition and indoor positioning. Furthermore, an experimental testbed for validating the effectiveness and feasibility of the smart home system was built and verified experimentally. The results showed that the 3D gesture recognition algorithm could achieve recognition rates for automated household appliance control of 92.0%, 94.8%, 95.3%, and 87.7% by the 2-fold cross-validation, 5-fold cross-validation, 10-fold cross-validation, and leave-one-subject-out cross-validation strategies. For indoor positioning and smart energy management, the distance accuracy and positioning accuracy were around 0.22% and 3.36% of the total traveled distance in the indoor environment. For home safety and fire detection, the classification rate achieved 98.81% accuracy for determining the conditions of the indoor living environment.

## 1. Introduction

According to the World Health Organization (WHO) Global Health and Aging report, approximately 524 million people, representing 8% of the world’s population, were aged 65 or older in 2010. By 2050, this number is estimated to reach 1.5 billion (around 16% of the world’s population) [1]. In addition, the WHO estimates that 650 million people live with disabilities around the world [2,3]. Therefore, it is necessary for researchers to develop user-friendly smart environments for promoting a better quality of life for elderly and disabled people. Smart homes utilize context-aware and location-aware technologies to create an intelligent automation and ubiquitous computing home environment for comfort, energy management, and safety and security [4,5,6,7].

Recently, a number of researchers have developed diverse technologies for smart homes, such as internet of things (IoT), intelligent control, home automation, energy management, and wearable devices [3,8,9,10,11,12,13,14,15]. In addition, the functions of remote control of household appliances, smart energy management, and indoor condition monitoring can be used to provide a comfortable and safe environment for residents. Hence, in this paper we propose a smart home system incorporating wearable intelligent technology, artificial intelligence, and multisensor data fusion technology, which can control household appliances remotely using an inertial-sensing-based gesture recognition algorithm, locate residents’ position in the indoor environment using an inertial- sensing-based indoor positioning algorithm, and determine the environmental conditions of the living spaces using an intelligent fire detection and alarm algorithm, for implementing features such as home automation control, smart energy management, and home safety. Recent studies have shown that inertial-sensing-based wearable devices have become a popular solution for gesture recognition and indoor location applications [16,17,18,19,20]. To name a few, Hsu et al. [21] developed an inertial-sensor-based pen with a dynamic time warping (DTW) recognizer for categorizing 800 samples collected from ten subjects. The recognition rates for recognizing eight 3-dimensional (3D) gestures reached 98.1% and 99.8% for user-independent and user-dependent recognition, respectively. Wang and Chuang [22] presented an accelerometer-based digital pen with a trajectory recognition algorithm for gesture trajectory recognition, which consists of time-domain and frequency-domain feature generation, a kernel-based class separability-based feature selection method, a linear discriminant analysis (LDA)-based feature reduction method, and a probabilistic neural network (PNN)-based classifier. The overall gesture recognition rate was 98.75% using a total of 800 samples with eight gestures. Hong et al. [23] proposed a motion gesture recognition system based on accelerations for classifying confusion set and easy set gestures, which extracted time-domain, frequency-domain, and singular value decomposition (SVD) based features, selected significant features using the mRMR approach, and classified gestures using the support vector machine (SVM) classifier. The accuracy for categorizing the confusion set and easy set gestures were 89.92% and 95.40%, respectively.

In terms of indoor positioning, Li et al. [24] designed an indoor positioning system by integrating Bluetooth beacons and a pedestrian dead reckoning (PDR) technique to provide indoor positioning without additional infrastructure. Likewise, Tian et al. [25] proposed a pedestrian tracking system using dead reckoning enhanced with a mode detection, which comprises mode detection, step detection, step length estimation, and orientation determination. Ren et al. [26] used a hidden Markov model for zero velocity detection and a Kalman filter (KF) for sensor, attitude, velocity, and position error estimation to develop a foot-mounted inertial-sensor-based pedestrian navigation system, while Hsu et al. [27] presented a wearable inertial pedestrian navigation system and its associated pedestrian trajectory reconstruction algorithm for pedestrian localization, which utilized a double-stage quaternion-based extended Kalman filter (EKF) to fuse accelerations, angular velocities, and magnetic signals for estimating the walking heading angle accurately.

With regard to intelligent fire detection and home safety, Rose-Pehrsson et al. [28] utilized a four smoke sensor array and a PNN to develop an early warning fire detection system for enhanced detection precision. Derbel [29] used gas sensors with its fire detection algorithm comprising a pre-processing unit, a fast Fourier transform (FFT)-based feature extraction unit to find significant fire features, and a learning vector quantization (LVQ) neural network to classify the significant features to fire, not fire, or disturbing event. Lee and Lee [30] utilized an Atmel AT89C51CC01 microcontroller to act as a controller area network (CAN) controller which collects the data measured by the smoke and gas sensors to form a network-based fire detection system for a smart home. Andrew et al. [31] utilized the principal component analysis (PCA)-PNN scheme to reduce and classify the features extracted from the measurements of the gas sensors, dust particles, temperature sensors, and humidity sensors for classifying incipient stage fires in buildings. Luis et al. [32] integrated a CO sensor, a smoke sensor, temperature sensors, a microcontroller, a short-range radio transceiver, a battery, a capacitive touch button, a LED, and a buzzer into a novel sensing device and developed a fire detection algorithm for home fire detection in indoor environments.

Based on the abovementioned literature review, a self-developed and low-cost smart home system and its associated intelligent-based gesture recognition algorithm, indoor positioning algorithm, and fire detection and alarm algorithm is developed in this paper for the purpose to provide an intelligent automation and ubiquitous computing home environment. The proposed smart home system consists of a wearable inertial sensing module, a multisensor circuit module, an information processing module, a decision-making module, an intelligent monitoring interface, and a household appliances plant. The wearable inertial sensing module is used to detect motion signals generated by hand and foot movements for recognizing human gestures and positioning residents’ indoor locations for household appliances remote control and smart energy management functions, respectively. The multisensor circuit module integrates CO sensors and temperature sensors for monitoring indoor environment for home safety and fire detection. In order to validate the effectiveness and feasibility of the smart home system, an experimental testbed is built and confirmed experimentally. The objective of this study is to integrate the wearable intelligent technology, artificial intelligence, and multisensor data fusion technology to provide home automation, energy management, and home safety functions for a smart home.

The rest of this paper is organized as follows: in Section 2, the proposed smart home system and architecture are described in detail. The intelligent algorithms composed of a 3D gesture recognition algorithm, an indoor positioning algorithm, and an intelligent fire detection and alarm algorithm are described in Section 3. Section 4 presents the experimental results. Finally, the conclusions are given in Section 5.

## 2. Proposed Smart Home System and Architecture

**Figure 1:**
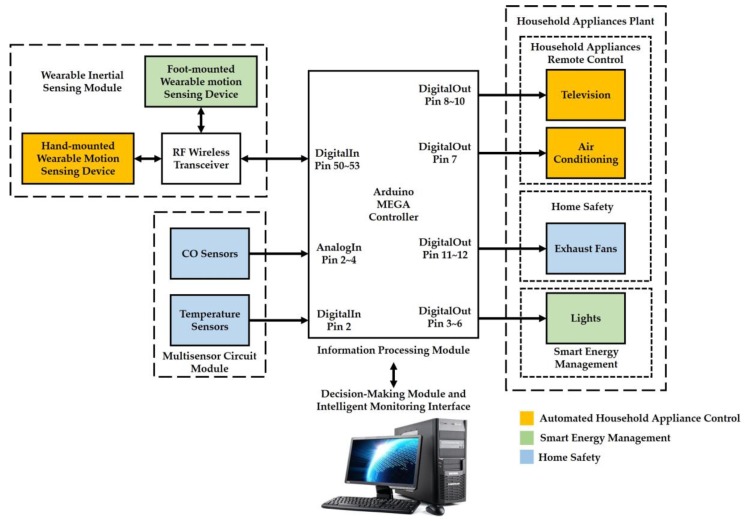
Overview system architecture of the proposed smart home system.

In this paper, we have implemented the following three functions to create an intelligent environment in the proposed smart home system: (1) automated household appliance control, (2) smart energy management, and (3) home safety. The architecture of the proposed smart home system consists of a wearable inertial sensing module, a multisensor circuit module, an information processing module (an Arduino MEGA microcontroller), a decision-making module (a personal computer, PC), an intelligent monitoring interface, and a household appliances plant. An overview of the system architecture for the smart home system is illustrated in Figure 1. The wearable inertial sensing module is used to detect motion signals generated by hand and foot movements for remote control of household appliances and smart energy management functions, respectively. The multisensor circuit module integrates CO sensors and temperature sensors for the function of home safety. The information processing module is responsible for connecting an RF wireless receiver through an SPI interface for collecting the motion signals measured from the wearable inertial sensing module, collecting the CO concentrations and temperatures measured from the multisensor circuit module through analog and digital pins, transmitting the abovementioned environmental measurements to the decision-making module for generating adequate decision commands through an universal series bus (USB) and to the intelligent monitoring interface for displaying the environmental conditions of the smart home in real-time. The decision-making module is utilized to develop a 3D gesture recognition algorithm, an indoor positioning algorithm, and an intelligent fire detection and alarm algorithm for determining the environmental conditions, generating the adequate decision commands, and sending the decision commands to the information processing module for further controlling the devices in the household appliances plan. The household appliances plant is used to receive the decision commands to turn on/off a television (TV) in the living room, an air conditioner in the living room, exhaust fans in the kitchen and bathroom, and lights in the living room, kitchen, bathroom, and corridor.

### 2.1. Wearable Inertial Sensing Module

**Figure 2:**
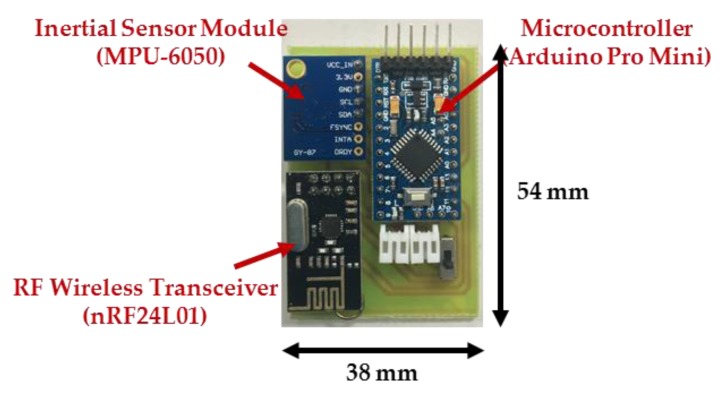
Wearable motion sensing device.

**Figure 3:**
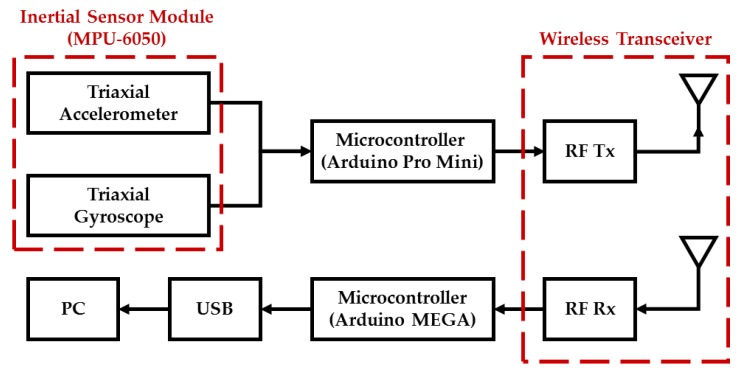
Schematic diagram of the wearable motion sensing device.

In this paper, we develop a wearable inertial sensing module which consists of a wearable motion sensing device mounted on residents’ wrists for detecting hand gesture motion signals and a wearable motion sensing device mounted on residents’ feet for capturing walking motion signals. The proposed wearable motion sensing device is composed of a microcontroller (Arduino Pro Mini, SparkFun Electronics, Boulder, CO, USA), a six-axis inertial sensor module (MPU-6050, InvenSense Inc., San Jose, CA, USA), an RF wireless transmission module (nRF24L01, SparkFun Electronics, Boulder, CO, USA), and a power supply circuit. The dimensions of the device are 54 mm × 38 mm × 10 mm, as shown in Figure 2. The Arduino Pro Mini microcontroller embedded in the wearable motion sensing device is responsible for collecting the digital signals measured by the six-axis inertial sensor module through an I^2^C interface and connecting to the RF wireless transceiver through an SPI interface. A six-axis inertial sensor which comprises a triaxial accelerometer, a triaxial gyroscope, and 16 bit analog to digital converters (ADCs) is utilized to simultaneously collect the accelerations and angular velocities generated by hand gestures and walking movements in a 3D space and output the digital measurement signal. The accelerometer can measure the gravitational and motion accelerations of hand gestures and walking motions, and possesses a user selectable full scale of ±2, ±4, ±8, and ±16 g. The gyroscope can detect the *X*-, *Y*-, and *Z*-axis angular velocities of the wearable motion sensing devices mounted on residents’ wrist and foot during making gesture and walking, and has a full scale of ±250, ±500, ±1000, and ±2000°/s. In this paper, the measurement range and sensitivity of the accelerometer are set as ±8 g and 4096 LSB/g, while those of the gyroscope are set as ±2000°/s and 16.4 LSB/°/s. The sampling rate of the measured inertial signals is 100 Hz. The accelerations and angular velocities are transmitted wirelessly to the information processing module (Arduino MEGA microcontroller, SparkFun Electronics, Boulder, CO, USA) via the RF wireless transceiver, which further sends them to the decision-making module (PC) for generating adequate decision commands and displaying the environmental conditions of the smart home in the intelligent monitoring interface. The power supply circuit provides the power consumption for the wearable motion sensing device, which is composed of a Li-ion battery, a Li-ion battery charging module, and regulators. The overall power consumption of the hardware device is 57 mA at 3.7 V. A schematic diagram of the wearable motion sensing device is shown in Figure 3.

### 2.2. Environmental Sensors

To implement the function of home safety, we installed temperature sensors (DS18B20) and carbon monoxide (CO) sensors (MQ-7) in the living room, bathroom, and kitchen to form a multisensor circuit module that monitors the indoor environmental temperatures and CO concentrations. The temperature sensors produced by Dallas Semiconductor Company (Dallas, TX, USA) output the digital measurement signals. The measurement range and accuracy of the temperature sensor are −55 °C~+125 °C and ±2 °C. The CO sensors produced by Hanwei Electronics Company (Henan, China) are composed of a tin dioxide (SnO_2_) gas sensing film, a micro Al_2_O_3_ ceramic tube, a heater, and measuring electrodes, and output analog measurement signals. The CO sensors can transduce CO concentrations to a resistance change with a measurement range of 20~2000 ppm, and possess a sensitivity of $1\%\;R/R_{o}/\mathrm{ppm}$ CO and a resolution limit of 20 ppm at low CO concentration. In addition, the CO sensors possesses the following features for indoor environmental monitoring: high sensitivity, a wide detection range, fast response time, long life, low cost, and simple drive circuits.

### 2.3. Experimental Testbed

**Figure 4:**
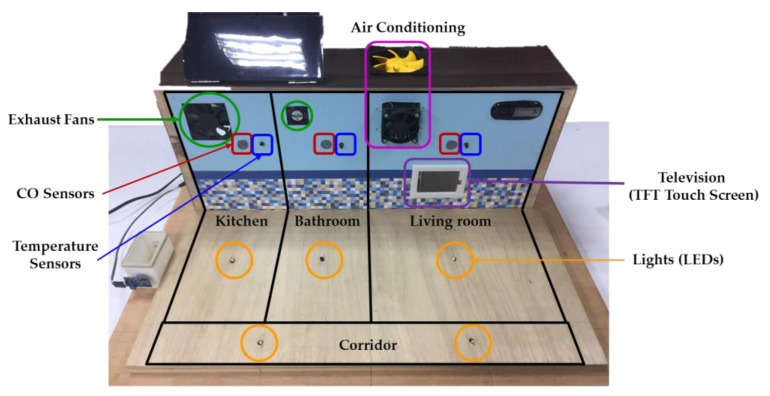
Experimental testbed of the smart home.

**Figure 5:**
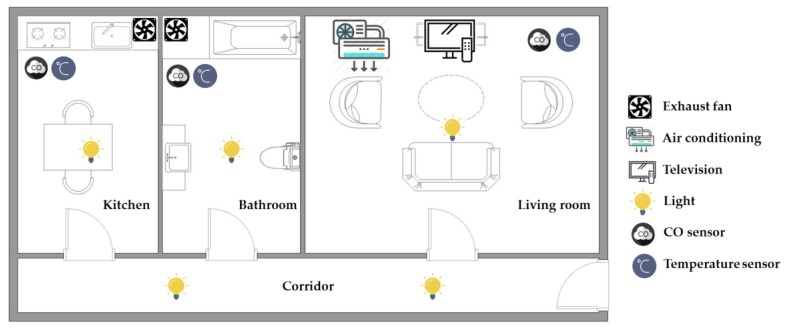
Layout of the smart home with environmental sensor location and placement.

The experiments were carried out in an experimental testbed measuring 50.9 cm × 44.0 cm × 23.3 cm (length × width × height), as shown in Figure 4. In the designed experimental testbed, a television (TFT touchscreen) and an air conditioner (a cooling chip combined with a radiator fan) are placed in the living room, while two exhaust fans are placed in the kitchen and bathroom, respectively. Three CO sensors and three temperature sensors are located in the living room, kitchen, and bathroom for detecting the indoor environmental temperatures and CO concentrations. Five lights (light-emitting diodes, LEDs) are installed in the living room, kitchen, bathroom, and corridor, respectively. The layout of the smart home with sensor location and household appliance deployment is shown in Figure 5.

### 2.4. Intelligent Monitoring Interface

**Figure 6:**
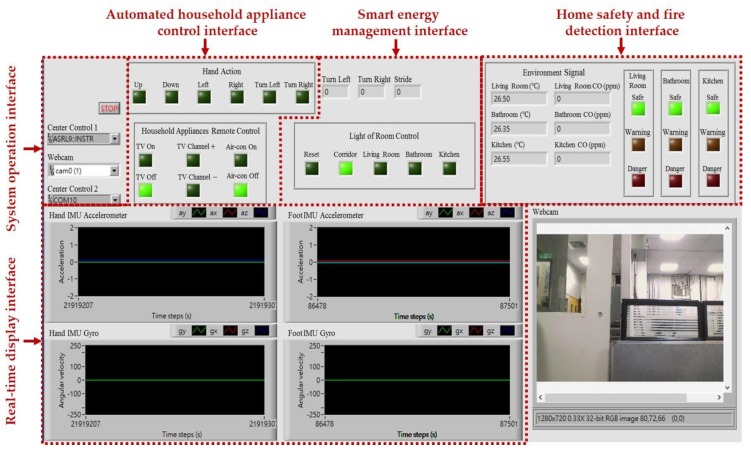
Intelligent monitoring interface for the smart home.

The intelligent monitoring interface in this paper shown in Figure 6 is developed by using the LabVIEW graphical programming environment, which can be divided into the following operation interfaces: (1) System operation interface: The operation interface includes a serial COM port selection button and a stop button. Residents can choose a serial COM port to receive the measurement signals and click the stop button to stop the signal analysis and display, respectively. (2) Real-time display interface: The real-time display interface can display filtered accelerations and angular velocities generated by hand gesture and walking motions in real-time, which can be measured by the wearable motion sensing devices mounted on residents’ wrist and foot, respectively. (3) Automated household appliance control interface: The interface shows the results of the gesture recognition and the present statuses of the household appliances in the living room. (4) Smart energy management interface: The interface shows the results of the indoor positioning and the present statuses of the lights located in the living room, kitchen, bathroom, and corridor. (5) Home safety and fire detection interface: The interface shows the temperatures and CO concentrations in the indoor environment, the results of the intelligent fire detection and alarm algorithm, and the present conditions of the living room, kitchen, and bathroom.

## 3. Proposed Intelligent Algorithms for the Smart Home

In this paper, we have implemented the following three intelligent algorithms to create an intelligent environment in the smart home: (1) A 3D gesture recognition algorithm to implement a real-time, convenient, and low-cost household appliances remote control system. (2) An indoor positioning algorithm to realize an effective indoor pedestrian navigation system for smart energy management. (3) An intelligent fire detection and alarm algorithm to realize an intelligent fire detection and alarm system for home safety and fire detection. We now introduce the detailed procedures of the proposed intelligent algorithms.

### 3.1. 3D Gesture Recognition Algorithm for Automated Household Appliance Control

**Figure 7:**
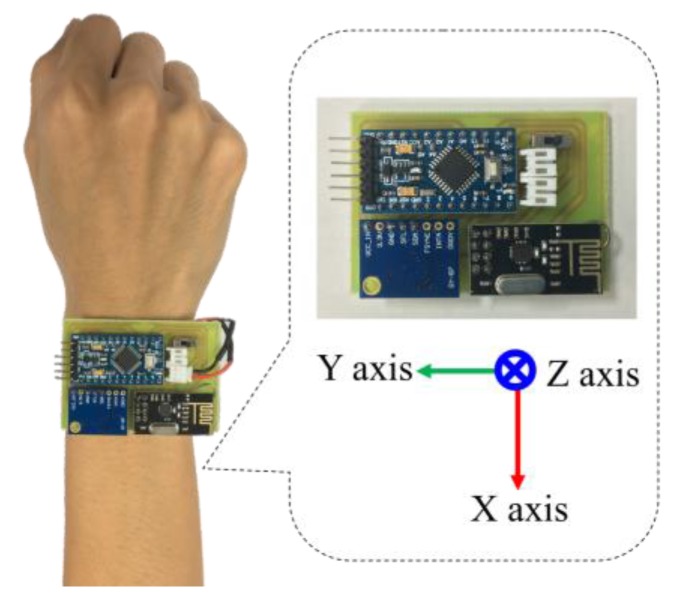
Wearable motion sensing device mounted on wrist.

**Figure 8:**
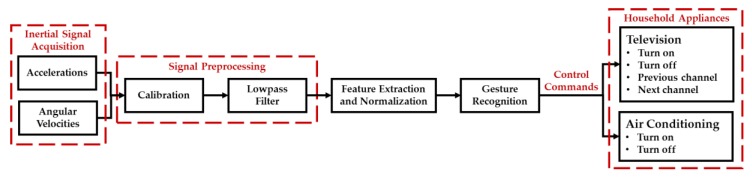
Block diagram of the 3D gesture recognition algorithm.

A 3D gesture recognition algorithm has been developed in this study to deal with hand gesture motion signals measured by the wearable motion sensing device mounted on residents’ wrists (Figure 7) for implementing the automated household appliance control function. Residents can utilize the wearable motion sensing device to make hand gestures at their preferred speed without any space limitations for generating the adequate decision commands to remotely control the household appliances. The proposed 3D gesture recognition algorithm is composed of the procedures of: (1) inertial signal acquisition, (2) signal preprocessing, (3) feature extraction and normalization, and (4) gesture recognition. First, the accelerations and angular velocities are collected by the microcontroller embedded in the wearable device and then transmitted to the information processing module (Arduino MEGA microcontroller, SparkFun Electronics, Boulder, CO, USA) and decision-making module (PC) via the RF wireless transceiver (nRF24L01, SparkFun Electronics, Boulder, CO, USA). Second, a calibration process and a designed lowpass filter are used to eliminate the sensitivity and offset errors of the sensors and residents’ unconscious trembles in the signal preprocessing procedure. Third, eight features are extracted from the accelerations and angular velocities to be as the inputs of the gesture recognizer for classifying six types of gestures. Finally, the recognition results are processed through a probabilistic neural network (PNN) and then generate the operational commands to remote control the household appliances. The block diagram of the proposed 3D gesture recognition algorithm is shown in Figure 8 and introduced in detail as follows.

#### 3.1.1. Signal Preprocessing

Once the inertial signals are acquired, the signal preprocessing procedure composed of calibration and lowpass filtering is an important procedure for reducing the efforts of the sensors’ error sources and removing residents’ unconscious trembles. First, we utilize the calibration method proposed in [33,34] to obtain the scale factor (SF) and offset (O) of each axis of the accelerometer and gyroscope, respectively, which are used to calibrate the measurements of the inertial sensors as Equation (1):

(1) $$M_{c}=SF\times M_{r}+O,$$

where $SF=\left[\begin{array}{ccc} SF_{x} & 0 & 0\\ 0 & SF_{y} & 0\\ 0 & 0 & SF_{z} \end{array}\right]$ and $O={\left[\begin{array}{ccc} O_{x} & O_{y} & O_{z} \end{array}\right]}^{T}$ represent the scale factor and offset of the triaxial accelerometer or gyroscope.$\;M_{c}$ is the calibrated accelerations ($A_{c}={\left[\begin{array}{ccc} a_{cx} & a_{cy} & a_{cz} \end{array}\right]}^{T}$) or angular velocities ($\omega_{c}={\left[\begin{array}{ccc} \omega_{cx} & \omega_{cy} & \omega_{cz} \end{array}\right]}^{T}$). $M_{r}$ is the raw accelerations ($A_{r}={\left[\begin{array}{ccc} a_{rx} & a_{ry} & a_{rz} \end{array}\right]}^{T}$) or angular velocities ($\omega_{r}={\left[\begin{array}{ccc} \omega_{rx} & \omega_{ry} & \omega_{rz} \end{array}\right]}^{T}$) before the calibration procedure. Secondly, we design a moving average filter to reduce the high-frequency noise of the calibrated accelerations or angular velocities and then the filtered accelerations $\left(A_{l}={\left[\begin{array}{ccc} a_{lx} & a_{ly} & a_{lz} \end{array}\right]}^{T}\right)$ and angular velocities $\left(\omega_{l}={\left[\begin{array}{ccc} \omega_{lx} & \omega_{ly} & \omega_{lz} \end{array}\right]}^{T}\right)$ can be obtained.

#### 3.1.2. Feature Extraction and Normalization

Once the filtered triaxial accelerations and angular velocities of each gesture are obtained, the gesture features can be extracted from the $a_{lx}$, $a_{ly}$, $a_{lz}$, $\omega_{lx}$, $\omega_{ly}$, and $\omega_{lz}$, respectively. The gesture features composed of: (1) minimum, (2) maximum, (3) mean, (4) standard deviation, (5) variance, (6) interquartile range, (7) root mean square, and (8) mean absolute deviation are used for recognizing the hand gesture patterns. More detailed information for the proposed gesture features can be found in [22]. Subsequently, the Z-score method is utilized to normalize each feature for eliminating the effects of the variation in the range of values of the gesture features, which may decrease the recognition rate [35].

#### 3.1.3. Gesture Recognition

**Figure 9:**
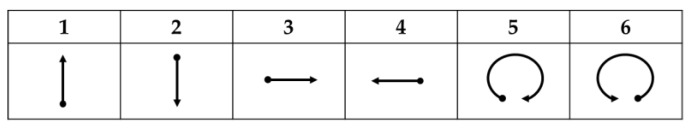
Patterns of six hand gestures.

**Figure 10:**
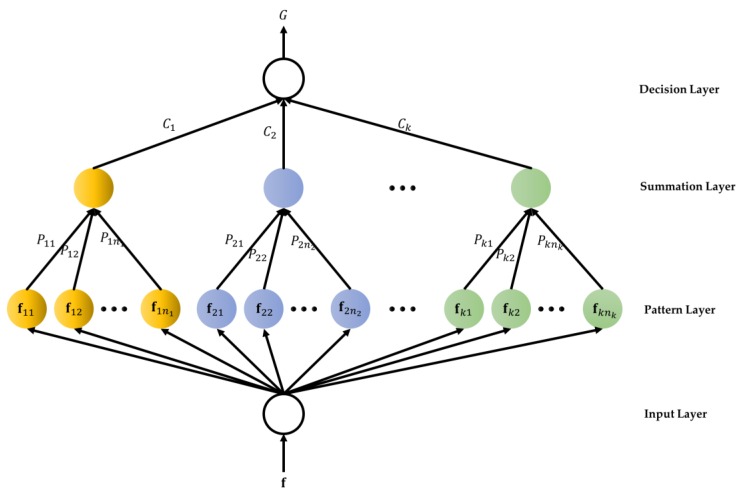
Structure of the probabilistic neural network recognizer.

After the feature extraction and normalization procedure, the normalized gesture features are used as input features for a PNN recognizer. The PNN recognizer can divide the hand gestures into: (1) swing upwards, (2) swing downwards, (3) swing right, (4) swing left, (5) circle, and (6) anti-circle for performing turn on TV, turn off TV, next TV channel, previous TV channel, turn on air conditioning, and turn off air conditioning, respectively, which are shown in Figure 9 and the gesture-referent mapping is described in Table 1. The PNN based on Baye’s strategy is developed to deal with recognition or classification problems [36]. The structure of the PNN classifier shown in Figure 10 consists of an input layer, a pattern layer, a summation layer, and a decision layer, and the training rule is based on the probability density functions of the classes. The neurons of the input layer convey the normalized gesture features $f={\left[f_{1},f_{2},\ldots ,f_{n}\right]}^{T}$ to the neurons in the pattern layer directly, where *n* is the number of the normalized features. In the pattern layer, the output of the neuron ($P_{kp}$) is derived by the multi-dimensional Gaussian function with the input pattern vector f conveyed from the input layer:

(2) $$P_{kp}=\frac{1}{{\left(2\pi \right)}^{n/2}\sigma^{n}}\exp\left[-\frac{{\left(f-f_{kp}\right)}^{T}\left(f-f_{kp}\right)}{2\sigma^{2}}\right],$$

where $f_{kp}$ is the neuron vector, and σ is the smoothing parameter. The neuron calculates the maximum likelihood of the input features (f) belonging to the class k. The neurons in the summation layer calculate the maximum likelihood of the pattern vector f that belong to the same class by averaging the outputs of all pattern layer neurons:

(3) $$C_{k}=\frac{1}{{\left(2\pi \right)}^{n/2}\sigma^{n}}\frac{1}{n_{k}}\sum_{i=1}^{n_{k}}\exp\left[-\frac{{\left(f-f_{kp}\right)}^{T}\left(f-f_{kp}\right)}{2\sigma^{2}}\right],$$

where $n_{k}$ is the total number of the gestures in class k. The neuron in the decision layer compares the outputs of all neurons in the summation layer and decides the class numerical label:

(4) $$G=\arg\;\max\;C_{k},\;k=1,2,\ldots ,N_{c},$$

where G denotes the estimated class and $N_{c}$ is the number of the classes. In this paper, the output of the PNN classifier can be labeled as ‘1’, ’2’, ‘3’, ‘4’, ‘5’, and ‘6’ which are represented as (1) swing upwards, (2) swing downwards, (3) swing right, (4) swing left, (5) circle, and (6) anti-circle, respectively. Finally, the abovementioned recognized gestures can be transformed to the following control commands for remotely controlling the household appliances: (1) turn on TV, (2) turn off TV, (3) next TV channel, (4) previous TV channel, (5) turn on air conditioning, and (6) turn off air conditioning, respectively.

### 3.2. Indoor Positioning Algorithm for Smart Energy Management

**Figure 11:**
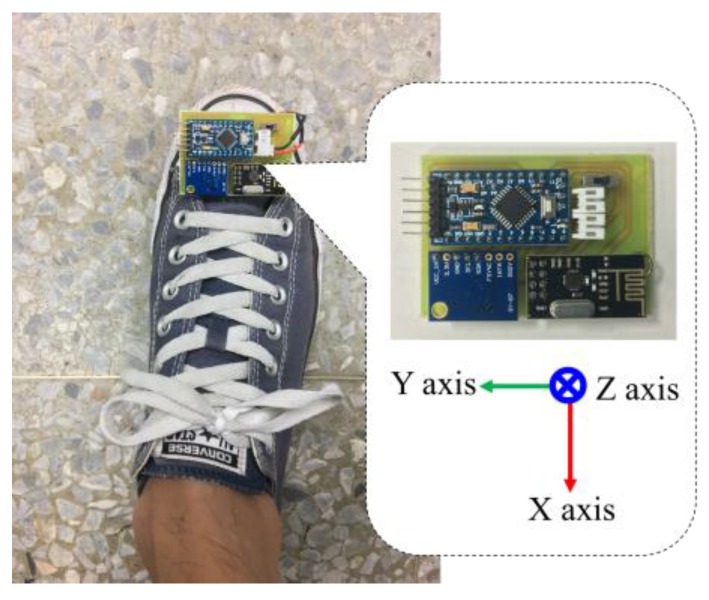
Wearable motion sensing device mounted on foot.

**Figure 12:**
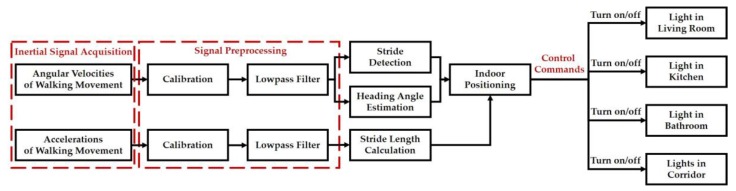
Block diagram of the indoor positioning algorithm.

An indoor positioning algorithm has been developed in this study to deal with walking motion signals measured from the wearable motion sensing device mounted on the residents’ feet (Figure 11) for implementing the smart energy management function. Residents can utilize the wearable motion sensing device to walking at their preferred speed without any space limitations for estimating walking trajectory and generating the adequate decision commands to remotely control the lights located in the living room, kitchen, bathroom, and corridor, respectively. The proposed indoor positioning algorithm is composed of the procedures of: (1) inertial signal acquisition, (2) signal preprocessing, (3) stride detection, (4) heading angle estimation, (5) stride length calculation, and (6) indoor positioning. First, the accelerations and angular velocities of walking movements are collected by the microcontroller embedded in the wearable device and then transmitted to the information processing module and decision-making module via the RF wireless transceiver. Second, a calibration process and a designed lowpass filter are used to eliminate the sensitivity and offset errors of the sensors, residents’ unconscious trembles, and walking friction in the signal preprocessing procedure. Third, the number of strides and the start and end points of each stride can be detected through setting a magnitude threshold of the filtered angular velocities. Simultaneously, the heading angle of the foot-mounted wearable inertial sensing device can be estimated through the integration of the filtered *Z*-axis angular velocity. Next, the stride length is calculated by the filtered walking accelerations. Finally, the indoor positioning or walking trajectories can be estimated based on the number of strides, walking heading angle, and individual stride length, and then generate the operational commands to remotely control the lights for performing the task of smart energy management. The block diagram of the proposed indoor positioning algorithm is shown in Figure 12 and introduced in detail as follows.

#### 3.2.1. Signal Preprocessing

Once the accelerations and angular velocities generated from walking movements are acquired, the signal preprocessing procedure composed of calibration and lowpass filtering is utilized to reduce the efforts of the sensors’ error sources and removing residents’ unconscious trembles and walking friction, which is the same as that in the proposed 3D gesture recognition algorithm.

#### 3.2.2. Stride Detection

A walking stride can be divided into two periodic periods: a static phase and a dynamic phase [37]. In this paper, we adopt a magnitude threshold method proposed in [34] to detect the start and end points of the dynamic phase using the signal vector magnitude (SVM) of the filtered angular velocities, which is defined as follows:

(5) $$\omega_{svm}=\sqrt{\omega_{lx}^{2}+\omega_{ly}^{2}+\omega_{lz}^{2}}.$$

**Figure 13:**
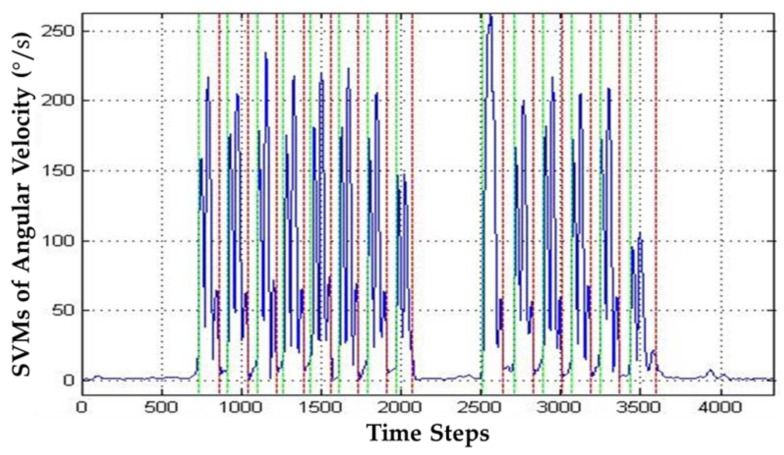
SVMs of the filtered angular velocities generated from walking movement. (Green color: Start points; Red color: End points).

Obviously, the SVM of the filtered angular velocities within the dynamic phase changes dynamically, while that within the static phase approximates to zero. In this paper, an empirical magnitude threshold for the SVM of the filtered angular velocities is set at 0.1°/s to the start and end points of the dynamic phase. More detailed information for the proposed stride detection method can be found in [34]. The start and end points of each dynamic phase of each stride determined by the magnitude threshold method are shown in Figure 13. Once the start and end points of each dynamic phase can be obtained, the number of strides can be calculated. That is, the pair of the start and end points is equal to the number of the strides.

#### 3.2.3. Heading Angle Estimation

In this paper, we estimate the heading angle of each start point of each stride through the single integral of the filtered *Z*-axis angular velocity measurement. The heading angle of the walking trajectory can be derived by the following equation:

(6) $$\theta_{t}=\theta_{t-1}+\frac{1}{2}\Delta T\left(\omega_{lz,t}+\omega_{lz,t-1}\right),$$

where t and t−1 represent the present and preceding time steps, ΔT is the sampling time, $\omega_{lz}$ is the filtered *Z*-axis angular velocity, and θ is the estimated heading angle.

#### 3.2.4. Stride Length Calculation

In the proposed indoor positioning algorithm, the more accurate the stride length calculation is, the more accuracy of the indoor positioning or walking trajectories we can obtain. In general, the human stride length is dependent on the personal gait characteristics. Hence, we calculate the stride length for each resident to obtain more accurate indoor positioning or walking trajectories using the stride length estimation method presented in [38]:

(7) $$SL_{k}=\alpha \sqrt[{4}]{a_{lz,k}^{max}-a_{lz,k}^{min}},$$

where $SL_{k}$ presents the estimated stride length of the *k*th stride, $a_{lz,k}^{min}$ and $a_{lz,k}^{max}$ are the minimal and maximal filtered *Z*-axis accelerations in the *k*th stride, and α is a constant.

#### 3.2.5. Indoor Positioning

Once the number of strides, walking heading angle, and stride length are obtained from the abovementioned procedures, the indoor pedestrian position can be estimated by the following equation:

(8) $$P_{k}=P_{k-1}+{\left[SL_{k}\begin{array}{cc} \cos\left(\theta_{k}\right), & SL_{k}\sin\left(\theta_{k}\right) \end{array}\right]}^{T},$$

where  k and k−1 represent the present and previous strides, $P={\left[\begin{array}{cc} P_{x}, & P_{y} \end{array}\right]}_{}^{T}$ is the estimated indoor pedestrian position, $\theta_{k}$ is the estimated heading angle of the present stride, and $SL_{k}$ is the stride length of the present stride. Finally, the control commands are generated to remotely turn on/off the lights placed in the living room, kitchen, bathroom, and corridor based on the present indoor location of the resident.

### 3.3. Intelligent Fire Detection and Alarm Algorithm for Home Safety

**Figure 14:**
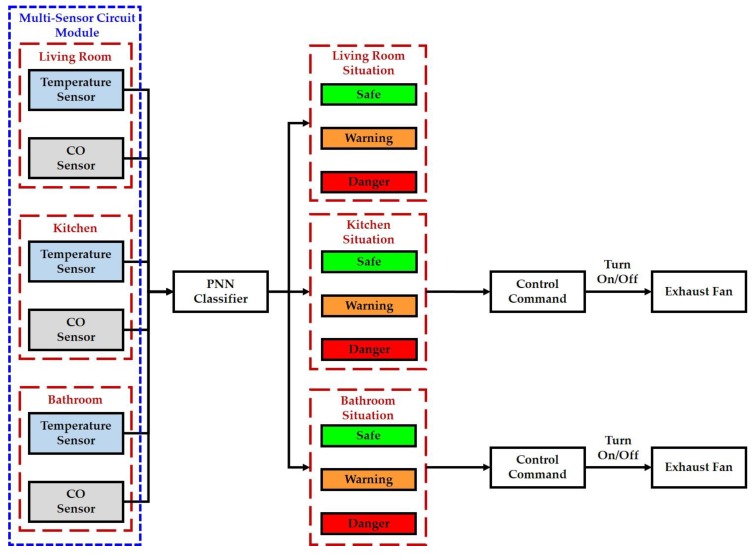
Block diagram of the intelligent fire detection and alarm algorithm.

An intelligent fire detection and alarm algorithm has been presented in this study to deal with the temperatures and CO concentrations measured by the temperature sensors (DS18B20, Dallas Semiconductor Company, Dallas, TX, USA) and CO sensors (MQ-7, Hanwei Electronics Company, Henan, China) installed in the living room, bathroom, and kitchen for implementing the home safety function. First, the temperature and CO concentration in each indoor environment are collected by the information processing module. Next, the multisensor data is transmitted from the information processing module to the decision-making module via the USB. Third, the abovementioned multisensor data can be as the inputs of a PNN classifier designed for determining the conditions of the living room, bathroom, and kitchen, which is divided into safe, warning, and danger. The structure of the PNN classifier is shown in Section 3.1.3. In this paper, the output of the PNN classifier can be labeled as ‘1’, ’2’, ‘3’, ‘4’, ‘5’, ‘6’, ‘7’, ‘8’, and ‘9’ which are represented as the safe, warning, and danger conditions of the living room, bathroom, and kitchen, respectively. Further, the proposed intelligent fire detection and alarm algorithm generates the control commands to turn on the exhaust fans placed in the kitchen and bathroom when the conditions of them are warning or danger, respectively. The block diagram of the proposed intelligent fire detection and alarm algorithm is shown in Figure 14.

## 4. Results

In this section, the effectiveness of the proposed 3D gesture recognition algorithm, indoor positioning algorithm, and intelligent fire detection and alarm algorithm is validated via the experimental results of household appliances remote control, indoor positioning and smart energy management, and home safety and fire detection in the indoor environment of the experimental testbed, respectively. All human materials such as human gesture motion and walking signals used in this study were approved by Institutional Review Board (IRB) of the National Cheng Kung University Hospital (IRB No. B-BR-102-032).

### 4.1. Automated Household Appliance Control

This experiment was designed to demonstrate the effectiveness of the proposed 3D gesture recognition algorithm for recognizing hand gestures and controlling household appliances. In this experiment, we collected gesture motion signals from ten subjects (three females, seven males; aged 21.3 ± 1.18 years old) in an indoor environment. The participants were asked to wear the wearable motion sensing device on their wrist and make six hand gestures in a 3D space, which are shown Figure 9. Each participant was invited to perform six hand gestures, and each gesture was to be making 10 times for this experiment. Hence, a total of 600 (=10 × 6 × 10) data were collected for this experiment. The best recognition rate achieved (Table 2) was 87.7% accuracy by leave-one-subject- out cross-validation. The recognition rates obtained by 2-fold cross-validation, 5-fold cross-validation, 10-fold cross-validation, and leave-one-subject-out cross-validation strategies were 92.0%, 94.8%, 95.3%, and 87.7%, as shown in Table 3.

Once the hand gestures can be recognized to swing upwards, swing downwards, swing right, swing left, circle, or anti-circle, which can generate the control commands for remotely controlling the household appliances, such as turn on TV, turn off TV, next TV channel, previous TV channel, turn on air conditioning, and turn off air conditioning, respectively.

**Figure 15:**
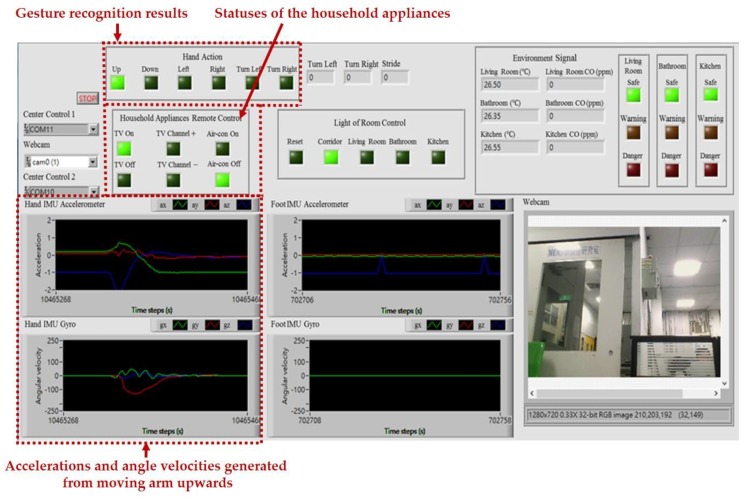
Accelerations and angular velocities generated from moving arm upwards, gesture recognition results, and statuses of the household appliances shown on the intelligent monitoring interface.

**Figure 16:**
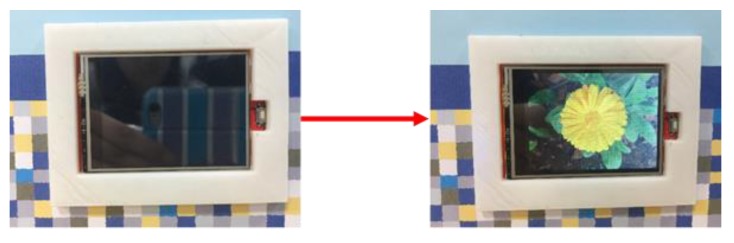
Turning on the TV via the swing upwards movement.

Figure 15 shows the intelligent monitoring interface which displays the acceleration and angular velocity signals generated from moving arm upwards (swing upwards), the gesture recognition results, and the statuses of the household appliances. The corresponding operation performed to turn on the TV is shown in Figure 16.

### 4.2. Indoor Positioning and Smart Energy Management

**Figure 17:**
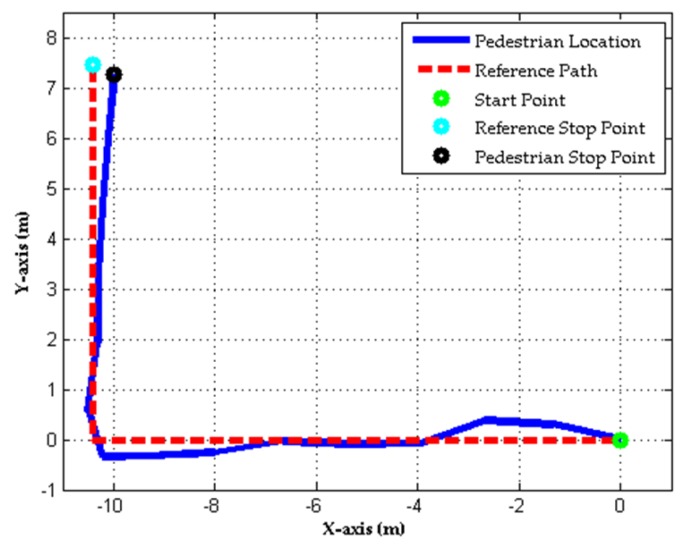
Estimated walking trajectory generated from the proposed indoor positioning algorithm.

This experiment was designed to demonstrate the effectiveness of the proposed indoor positioning algorithm for indoor pedestrian navigation and smart energy management. In this experiment, we used a walking path in an indoor environment to represent the path from the entrance to the living room in the experimental testbed for presenting the positioning results. The total distance of the walking path is about 17.82 m. In this experiment, we collected walking motion signals from the same subjects presented in the experiment of the household appliances remote control (three females, seven males; aged 21.3 ± 1.18 years old) in an indoor environment. Subjects were asked to mount the wearable motion sensing device on their foot and walking on the path at normal speed without any external localization techniques. For evaluating the accuracy of the estimated walking trajectories, the error of the distance ($e_{d}$), distance accuracy ($e_{da}$), end point error ($e_{e}$), and positioning accuracy ($e_{ea}$) can be computed as follows:

(9) $$e_{d}=\left|L_{r}-L_{e}\right|,$$

(10) $$e_{da}=\frac{e_{d}}{L_{r}},$$

(11) $$e_{e}=\sqrt{{\left(P_{x,re}-P_{x,ee}\right)}^{2}-{\left(P_{y,re}-P_{y,ee}\right)}^{2}},$$

(12) $$e_{ea}=\frac{e_{e}}{L_{r}},$$

where $L_{r}$ and $L_{e}$ are the total traveled distance of the walking path and estimated walking distance, $P_{x,re}$ and $P_{x,ee}$ are the *X*-coordinate position of the reference end point and estimated end point, $P_{y,re}$ and $P_{y,ee}$ are the *Y*-coordinate position of the reference end point and estimated end point. Figure 17 shows the estimated walking trajectory (pedestrian location) and actual path (reference path) in the indoor environment from one participant in particular. In the figure, the total traveled distance of the walking path ($L_{r}$) is about 17.82 m and the estimated walking distance ($L_{e}$) is 17.84 m. Therefore, the error of the distance ($e_{d}$) of the estimated walking trajectory using the indoor positioning algorithm was 0.04 m and its distance accuracy ($e_{da}$) was around 0.22% of the total traveled distance. In addition, the end point error ($e_{e}$) of the estimated walking trajectory using the indoor positioning algorithm was 0.59 m. Hence, the positioning accuracy ($e_{ea}$) was around 3.36% of the total traveled distance in the indoor environment. Once the resident’s location can be positioned in the living room, kitchen, bathroom, and corridor, which can generate the control commands for remotely controlling the switches of the lights placed in the living room, kitchen, bathroom, and corridor based on the present indoor location of the resident.

**Figure 18:**
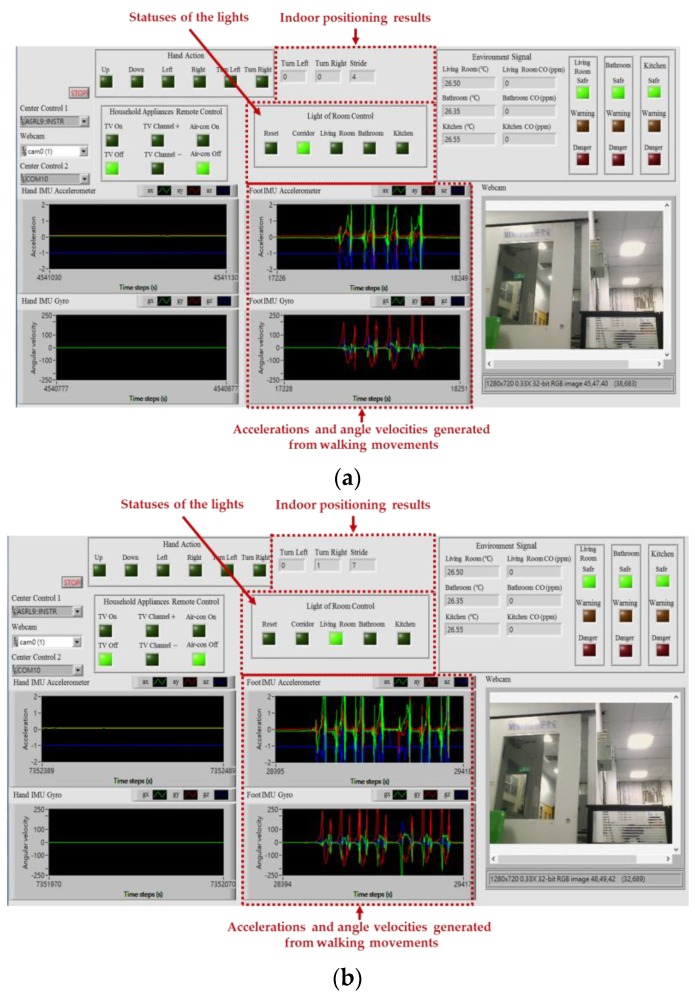
Accelerations and angular velocities generated from walking movements, indoor positioning results, and statuses of the lights shown on the intelligent monitoring interface. (**a**) The resident walked in the corridor, and the lights in the corridor were turned on and the others in the other indoor environment were turned off; (**b**) The resident left the corridor and walked into the living room, and the light in the living room was turned on and the others in the other indoor environment were turned off.

**Figure 19:**
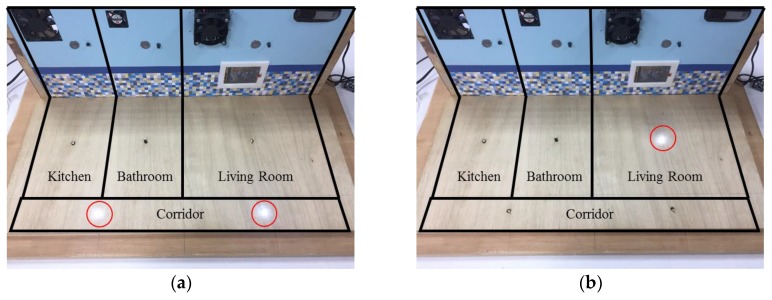
The statuses of the lights located in the experimental testbed when the resident left the corridor and walked into the living room based on the results shown in Figure 17. (**a**) Turn off the lights in the living room, kitchen, and bathroom, and turn on the lights in the corridor when the resident is in the corridor; (**b**) Turn off the lights in the kitchen, bathroom, and corridor, and turn on the lights in the living room when the resident is in the living room.

Figure 18 shows the intelligent monitoring interface which displays the acceleration and angular velocity signals generated from walking movements, the indoor positioning results, and the statuses of the lights based on the estimated walking trajectory shown in Figure 17. The corresponding operation performed to turn on the light in the living room and turn off the lights in the corridor when the resident left the corridor and walked into the living room is shown in Figure 19.

### 4.3. Home Safety and Fire Detection

**Figure 20:**
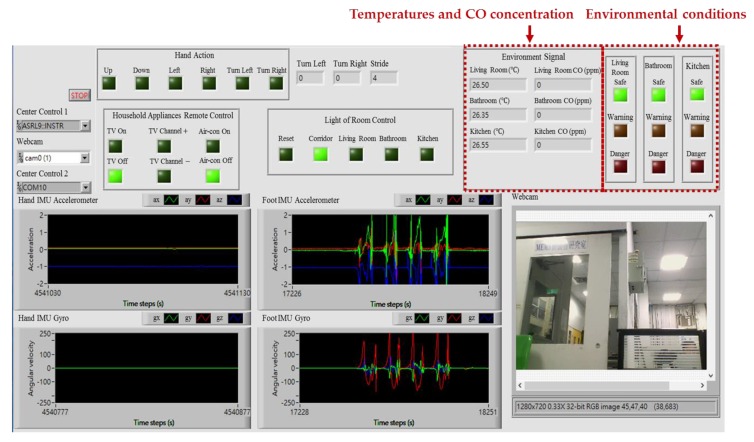
Temperatures, CO concentrations, and environmental conditions of the indoor environment shown on the intelligent monitoring interface.

This experiment was designed to demonstrate the effectiveness of the proposed intelligent fire detection and alarm algorithm for home safety and controlling the exhaust fans placed in the indoor environment. In this experiment, we installed the temperature sensors (DS18B20, Dallas Semiconductor Company, Dallas, TX, USA) and CO sensors (MQ-7, Hanwei Electronics Company, Henan, China) in the living room, bathroom, and kitchen for monitoring the temperatures and CO concentrations in the experimental testbed, respectively. A total number of 11,000 measurements were collected, 10,000 data for training the PNN classifier and 1000 data for testing the trained PNN classifier. The classification rate can achieve 98.81% accuracy. Once the environmental conditions of the living room, bathroom, and kitchen can be classified as safe, warning, or danger, this can generate the control commands for remotely controlling the switches of the exhaust fans placed in the kitchen and bathroom. Figure 20 shows the intelligent monitoring interface which displays the temperatures and CO concentrations and the classified conditions of the indoor environment.

**Figure 21:**
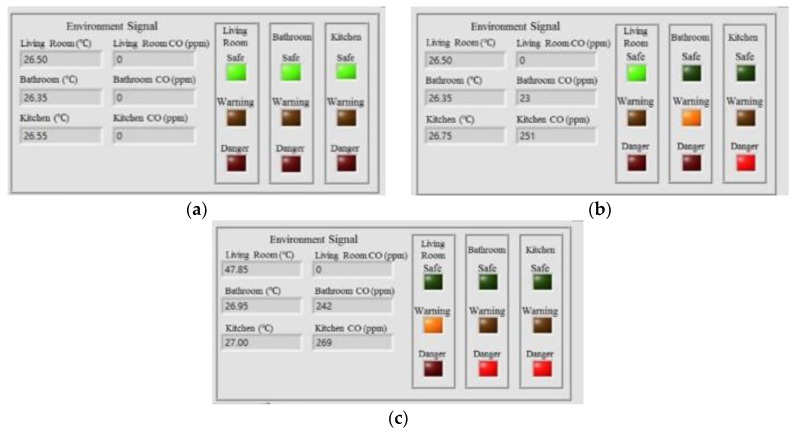
Experimental results of the home safety and fire detection. (**a**) The conditions of the living room, kitchen, and bathroom are all safe; (**b**) The living room is safe, kitchen is warning, and bathroom is danger; (**c**) The living room is warning, and kitchen and bathroom are both danger.

Figure 21 shows some experimental results to show the conditions of the indoor environment. Figure 21a shows that the conditions of the living room, bathroom, and kitchen are all safe, which are determined by the PNN with the temperature and CO concentration being 26.50 °C and 0 ppm in the living room, 26.35 °C and 0 ppm in the bathroom, and 26.55 °C and 0 ppm in the kitchen. Figure 21b shows the conditions of the living room, bathroom, and kitchen when the temperature and CO concentration are 26.50 °C and 0 ppm in the living room, 26.35 °C and 23 ppm in the bathroom, and 26.75 °C and 251 ppm in the kitchen. Figure 21c shows that the condition of the living room is warning, and the bathroom and kitchen are both danger when the temperature and CO concentration are 47.85 °C and 0 ppm in the living room, 26.95 °C and 242 ppm in the bathroom, and 27.00 °C and 269 ppm in the kitchen.

## 5. Conclusions

This study focused on developing a smart home system with wearable intelligent technology, artificial intelligence, and multisensor data fusion technology, which can control household appliances remotely, locate residents’ position in the indoor environment, and monitor the conditions of the living spaces to implement the functions of automated household appliance control, smart energy management, and home safety. The architecture of the proposed smart home system comprises a wearable inertial sensing module, a multisensor circuit module, an information processing module, a decision-making module, an intelligent monitoring interface, and a household appliances plant. For automated household appliance control, the 3D gesture recognition algorithm composed of inertial signal acquisition, signal preprocessing, feature extraction and normalization, and gesture recognition was developed for recognizing the hand gestures and then generated the commands to control the household appliances remotely. The recognition rates were 92.0%, 94.8%, 95.3%, and 87.7% for the 2-fold cross-validation, 5-fold cross-validation, 10-fold cross-validation, and leave-one-subject-out cross-validation strategies, respectively. For indoor positioning and smart energy management, the indoor positioning algorithm composed of inertial signal acquisition, signal preprocessing, stride detection, heading angle estimation, stride length calculation, and indoor positioning was developed for locating the residents’ position and then generated the commands to control the lights remotely. The error of the distance ($e_{d}$) and end point error ($e_{e}$) of the estimated walking trajectory using the indoor positioning algorithm were 0.04 m and 0.59 m. Therefore, the distance accuracy ($e_{da}$) and positioning accuracy ($e_{ea}$) were around 0.22% and 3.36% of the total traveled distance in the indoor environment, respectively. For home safety and fire detection, the classification rate was 98.81% for determining the conditions of the indoor living environment using the PNN classifier. In addition, the experimental testbed was built and confirmed experimentally for validating the effectiveness and feasibility of the smart home system. Based on the above experimental results, we believe that the proposed smart home system and its associated intelligent algorithms and functions will be provided a novel and effective contribution to smart home system design. In future studies, we intend to minimize the size of the wearable motion sensing device with improved wear comfortability and combine with other indoor positioning techniques, such as WiFi or Beacon, to improve the accuracy of indoor positioning. Moreover, we will implement the proposed 3D gesture recognition algorithm, indoor positioning algorithm, and intelligent fire detection and alarm algorithm on the Arduino microcontroller and develop a more convenient intelligent monitoring interface on a smartphone by using the Android programming environment for practical purposes.

## Figures and Tables

**Table 1:** Gesture-referent mapping.

Gesture	Description	Referents
Swing upwards	Move arm upwards	Turn on TV
Swing downwards	Move arm downwards	Turn off TV
Swing right	Move from left to right	Next channel
Swing left	Move from right to left	Previous channel
Circle	Draw clockwise circle	Turn on air conditioning
Anti-circle	Draw counter-clockwise circle	Turn off air conditioning

**Table 2:** Confusion matrix for 3D gesture recognition by leave-one-subject-out cross-validation.

Method	Swing Upwards	Swing Downwards	Swing Right	Swing Left	Circle	Anti-Circle
**Swing upwards**	87	1	3	2	4	3
**Swing downwards**	0	91	4	1	2	2
**Swing right**	6	5	86	3	0	0
**Swing left**	4	7	8	80	0	1
**Circle**	1	5	0	0	94	0
**Anti-circle**	1	10	1	0	0	88

R: Recognized, M: Made hand gestures.

**Table 3:** Recognition rates for 3D gesture recognition by validation methods.

Method	2-Fold	5-Fold	10-Fold	Leave-One-Subject-Out
**Recognition Rate**	92.0%	94.8%	95.3%	87.7%
